# Tracking Endometrial Malacoplakia Through the Evolution of 2D and 3D Ultrasound and Histopathological Features

**DOI:** 10.7759/cureus.52268

**Published:** 2024-01-14

**Authors:** Antonella Vimercati, Francesca Cirignaco, Rosalba De Nola, Marco Cerbone, Tommaso Difonzo, Pietro Quarto, Fabiana Divina Fascilla, Ettore Cicinelli, Leonardo Resta, Gerardo Cazzato

**Affiliations:** 1 Department of Precision and Regenerative Medicine and Ionian Area, University of Bari Aldo Moro, Bari, ITA; 2 Department of Interdisciplinary Medicin, University of Bari Aldo Moro, Bari, ITA; 3 Department of Interdisciplinary Medicine, University of Bari Aldo Moro, Bari, ITA; 4 Department of Obstetrics and Gynecology, Di Venere General Hospital, Bari, ITA; 5 Department of Pathology, University of Bari Aldo Moro, Bari, ITA

**Keywords:** michaelis–gutmann bodies, inflammatory pathology, ultrasound, endometrium, malacoplakia

## Abstract

Malacoplakia is an uncommon disease characterized by chronic and granulomatous inflammation, which rarely involves the female genital tract. We describe the ecographic and histological evolution of the first case of a patient developing endometrial malacoplakia as a complication after a cesarean section. The patient, a 43-year-old woman, presented with pelvic pain one month after delivering by cesarean section and the initial suspicion was of retention of placental rests. We discuss the diagnostic challenges for this rare disease, highlighting the importance of considering endometrial malacoplakia as a possible diagnosis in patients with similar clinical presentations and the important role of 2D and 3D ultrasound in the diagnostic pathway. In literature, ultrasound findings in cases of endometrial malacoplakia are represented by hypoechoic thickening of the endometrial lining; hyperechoic thickening of the myometrium, and the presence of masses, nodules, cystic areas, or anechoic fluid within the endometrium. For the first time, we describe the evolution of endometrial malacoplakia through both ultrasound, 2D and 3D, and histopathological findings, from the acute to chronic stage of the disease.

## Introduction

Malacoplakia is an infrequent chronic granulomatous inflammatory disease that can affect any part of the human body, with the genitourinary and gastrointestinal tracts being the most involved sites. It was first described by Michaelis et al. in 1902 and typically affects individuals over 50 years of age and is more commonly observed in women, with a female-to-male ratio of 4:1 [1]. The condition, like other maternal complications, is more likely to occur in patients with immunodeficiency disorders. Histologically, the disease is characterized by a mixture of inflammatory cells including lymphocytes, plasma cells, neutrophils, and histiocytes, some of which contain pathognomonic Michaelis-Gutmann (MG) bodies. There are approximately 500 cases of malacoplakia reported in medical literature to date. The majority of these cases involve the genitourinary tract, specifically the urinary bladder, kidney, ureter, or prostate. However, malakoplakia has also been reported outside the urinary tract, such as in the gastrointestinal tract, central nervous system, female genital tract, and tongue [2]. The disease's pathogenesis is thought to be caused by incomplete digestion of *Escherichia coli* by lysosomes, which occurs regardless of phagocytosis. This is likely due to defective lysosomal function, leading to the formation of von Hansemann cells, a characteristic histological finding in malakoplakia [3-14]. Involvement of the female genital tract is rare, with less than 40 cases reported to date, of which only 15 cases of endometrium malacoplakia [4-13,15-19].

## Case presentation

**Figure 1:**
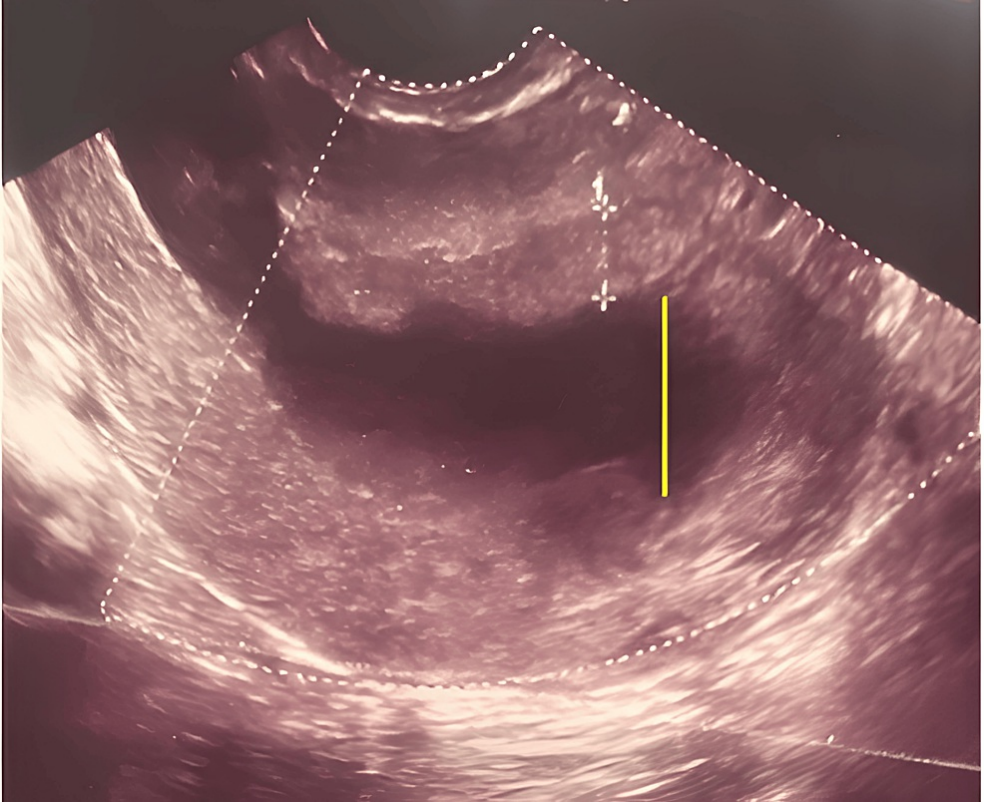
Transvaginal ultrasound, sagittal scan of the uterus. The endometrial cavity and cervical canal distended by anechoic endocavitary fluid collection with a maximum thickness of 26 mm (yellow line), clear thickened heterogeneous irregular endometrium of 7.8 mm, due to imbibition of the endometrial layer (white markers).

This report illustrates the case of endometrial malacoplakia in a 43-year-old patient readmitted to our hospital for pelvic pain one month following cesarean delivery. The patient was P 1011, with one previous cesarean section due to non-reassuring CTG (postnatally, the newborn had behavioral alterations and a difficult diagnosis of Pitt-Hopkins syndrome) and one spontaneous abortion. She was admitted to our hospital in July 2021 at 35 weeks with a diagnosis of reduction in fetal movement activity in the last 48 hours, mild fetal growth restriction (estimated fetal weight of 2190 g, corresponding to 8.7° percentile according to the WHO fetal growth calculator), oligohydramnios, absence of diastolic flow in the umbilical artery, and signs of centralization of the fetal circulation in the middle cerebral artery. The same day, a dose of 12 mg betamethasone was administered and later an urgent cesarean section was decided to perform due to non-reassuring CTG findings. During the cesarean section, after manual removal of the placenta, since uterine hypotonia occurred, both uterine massage and infusion of uterotonics were necessary to obtain resolution of the hypotonia. The newborn of the male sex had good well-being parameters at birth: Apgar: eight to 10; weight: 2340 g (AGA, appropriate for gestational age according to the Italian neonatal charts); and regular clinical course. The patient was discharged in July 2021. In August 2021, the patient reported mild pelvic pain and a gynecological examination was performed at a private medical institute. On this occasion, the ultrasound scan revealed the presence of an endocavitary fluid collection distending the endometrial cavity and cervical canal, with a maximum breadth of 26 mm and a clear heterogeneous thickened endometrium of 7.8 mm (Figure 1).

**Figure 2:**
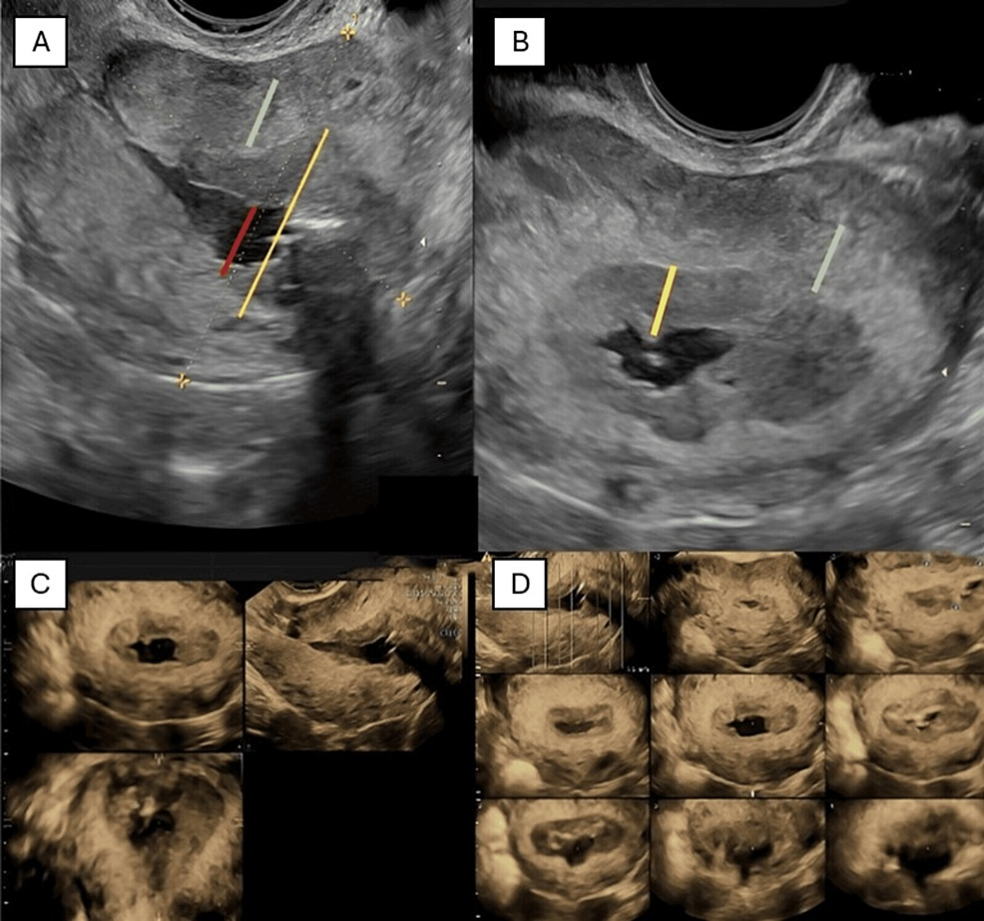
2D Transvaginal ultrasound sagittal (A) and axial (B) images of the uterus: endocavitary fluid collection with a maximum thickness of 10 mm (red line); hypoechoic endometrium (yellow line), irregular junctional zone, inhomogeneous myometrium with inner hyperechoic layer (green line); total breadth of the uterus affected by the inflammation process with imbibition of the endometrial tissue of 23 mm (yellow line). (C) 3D sagittal, axial, and coronal ultrasound scans: endocavitary fluid collection; inhomogeneous hypoechoic endometrial lining, inhomogeneous myometrium with hyperechoic inner myometrial. (D) 3D ultrasound in axial scan set on TUI mode, which simultaneously displays multiple slices of a volume data set: endocavitary fluid collection; hypoechoic endometrium, irregular junctional zone, inhomogeneous myometrium with inner hyperechoic layer. TUI, tomographic ultrasound imaging

A follow-up gynecological examination was recommended after 15 days and on this occasion, the new ultrasound exam revealed a heterogeneous endometrial thickening of 15 mm. Consequently, in the same institute two hysteroscopies were performed and biopsies were taken. The patient did not present the results of the gynecological ultrasound or the histological examination but revealed that solid white-yellow material was removed from the uterine cavity on that occasion. Due to intense pelvic pain, about four months after the cesarean section, in November, the patient was readmitted to our hospital. During the recovery, the ultrasound exam, using GE Healthcare Voluson S10, revealed a reduction in the size of the endocavitary fluid, a thickened hypoechoic endometrium, and a hyperechoic inner myometrium (Figure 2). The initial ultrasonographic image confirmed there was an endometrial abnormality, but the imaging appearance was nonspecific, demanding a differential diagnosis between common endometrial pathologies, such as retention of placental rests, inflammatory processes (endometritis), or post-surgical adhesions. The subsequent atypical evolution of the ultrasound findings, combined with suspicious signs and symptoms (persisting pain and fibrotic yellow vaginal secretions), led us to consider rare diseases, such as malakoplakia, in the differential diagnosis. We had reported similar ultrasound findings in a previous case but unfortunately have no documentation. Definitive diagnosis of malacoplakia requires histological examination.

Based on the ultrasound examination features, we suspected the presence of a chronic inflammatory process of the endometrium, demanding a differential diagnosis between an infectious etiology or other diseases such as endometrial malacoplakia. To our knowledge, this is the first case in literature where the ultrasound exam first aroused suspicion of endometrial malacoplakia, after excluding the presence of common endometrial pathologies. It is also the first case of endometrial malacoplakia representing a complication after a cesarean section. The patient had no known anamnestic risk factors, apart from her cesarean section, since she had no history of immunodeficiency conditions, such as steroid use, diabetes, or primary immunodeficiency. In order to confirm our suspicion and have a definitive histopathological diagnosis, we performed hysteroscopy with endometrial resection. Thick fibrotic white-yellowish layers, similar to skin rind, occupying the entire endometrial cavity, were removed.

**Figure 3:**
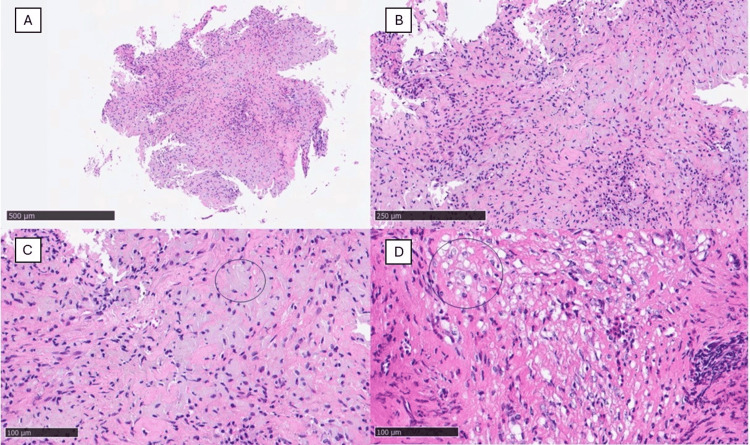
(A) Histological photomicrograph showing a biopsy of endometrial tissue with polymorphous elements (hematoxylin-eosin, original magnification 2x). (B) Histological photomicrograph showing histiocytic elements with abundant granular cytoplasm and basophilic inclusions (hematoxylin-eosin, original magnification 20x). (C) Scanning magnification of the previous picture shows the rich histiocytic component (an example is indicated by a black circle) intermingled between the tissue (hematoxylin-eosin, original magnification 40x). (D) Another picture in which eosinophils and macrophages predominate (hematoxylin-eosin, original magnification 40x).

The histological examination revealed the presence of infiltration of histiocytic elements with abundant granular cytoplasm and basophilic inclusions (so-called von-Hansemann cells), with accompanying polymorphous cells such as eosinophils, lymphocytes, plasma cells, and rare neutrophils (Figure 3). Furthermore, there were some areas of fibrosclerosis and no remnant chorionic tissue was observed. Immunochemical investigations revealed cells strongly tested positive for CD68 (PGM-1) and CD163 and negative for CK-pool.

**Figure 4:**
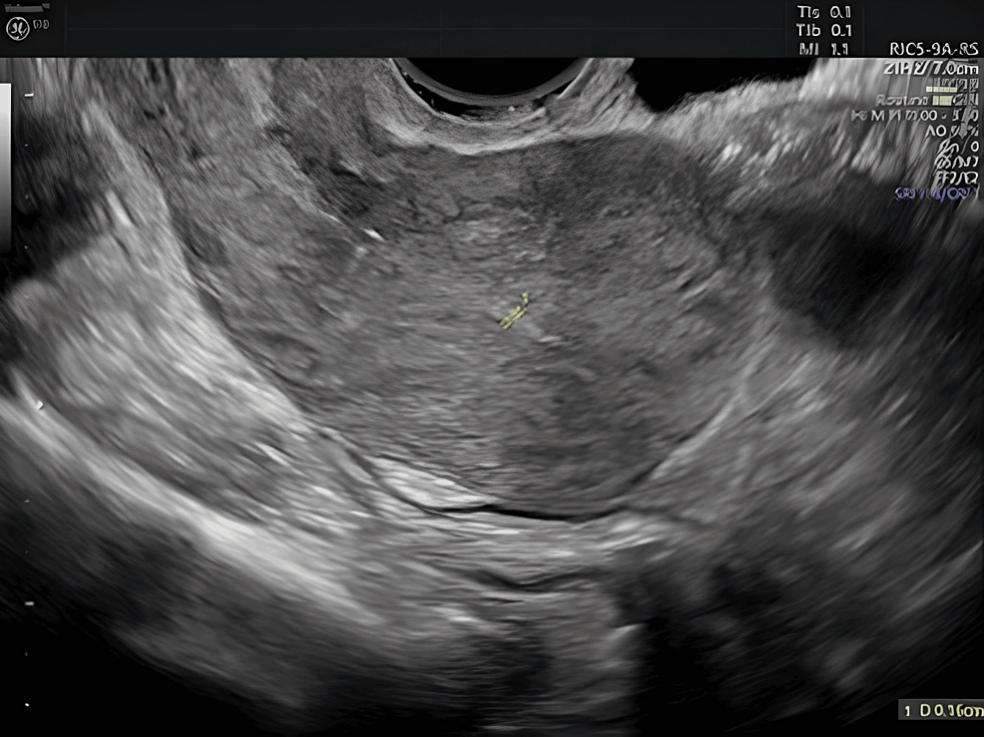
Transvaginal ultrasound sagittal scan of the uterus: thin linear homogeneous hyperechoic endometrium of 1.6 mm; regular homogeneous hypoechoic myometrium. Absence of endocavitary fluid.

After two months, a further resectoscopy with curettage was performed and the patient was treated with quinolone antibiotics, resulting in a rapid clinical improvement, complete resolution of symptoms, and a normal gynecological ultrasound report with the absence of endocavitary fluid (Figure 4).

## Discussion

While malacoplakia has been reported to affect various organs in the body, including the urinary tract, gastrointestinal tract, lungs, and skin, there are very few reported cases of malacoplakia specifically affecting the uterus. Most of the reported cases of malacoplakia in the literature are related to the genitourinary system, with the bladder being the most affected site. An increasing number of cases from more and more types of tissue have been reported in recent years. To our knowledge, up to the present time, there are less than 40 cases published describing the involvement of the female genital tract, 15 of these involving the endometrium, 13 the cervix, eight the vulva or vagina, three the ovary, and two the parametrium. The remaining cases involve two or more sites of the female genital tract. The most common presenting symptom is postmenopausal vaginal bleeding. Our case is the first presenting with persisting pelvic pain after surgery (cesarean section) and the absence of signs typical for other frequent endometrial pathologies, such as endometritis, post-surgery adhesions, endometriosis, or endometritis. Common causes of postmenopausal bleeding include hyperplasia, carcinoma of the endometrium, senile endometritis, endometrial polyps, postmenopausal hyperplasia, and pyometria. Even though rare, malacoplakia should be considered in the differential diagnosis of menstrual abnormalities. Other clinical findings are cervical mass, vaginal discharge, cervical ulceration, abdominal pain, or friable cervix [4]. Clinical suspicion of malacoplakia may be heightened by the appearance of vaginal secretions. In our case report solid white-yellow material was removed from the uterine cavity after curettage. Similar findings with endometrial fragments mixed with blood and yellowish nodular deposits are reported in the literature [5]. Typical pathological findings are thick, soft, gray-white endometrium, yellowish, nodular tissue aggregates, golden-yellowish plaques, and friable necrotic tissue. The endometrial mucosa is often thickened and multiple sites of focal hemorrhage can be found [5-7]. To our knowledge in literature, there are only seven cases of endometrium malacoplakia where ultrasound was performed and our case is the first to describe typical, although non-diagnostic, ultrasound features suspicious for this disease and track their evolution in various stages of the inflammatory process (Table 1). In all these cases reported the diagnosis for malacoplakia was made based on the histological findings.

**Table 1:** Summary of the previous cases reported in literature along with our case.

Reference	Patient age	Clinical presentation	Other pelvic structure involved	Ultrasound
[4]	78	Vaginal bleeding	Cervix	14 mm × 7 mm mass located in the posterior lip of the uterine cervix; no other pathological findings
[5]	69	Vaginal bleeding, pain in the left lower quadrant	No	Not performed
[6]	60	Postmenopausal bleeding	No	Not performed
[7]	47	Lower abdominal discomfort, urinary frequency, incontinence	Ovary, fallopian tube	Leiomyomas, right adnexal mass
[8]	40	Irregular menstrual bleeding	Broad ligament inguinal region	Not performed
[9]	71	Vaginal bleeding, pain in the left lower quadrant	Cervix	Not performed
[10]	72	Vaginal bleeding, pain in the lower abdominal wall	No	Not performed
[11]	88	Postmenopausal bleeding	No	Not performed
[12]	60	Vaginal bleeding	Cervix	Not performed
[13]	74	Vaginal bleeding	Cervix	Not performed
[14]	64	Mucoid, foul-smelling vaginal discharge	No	Enlarged uterus of 10.7 cm × 7.5 cm filled with linear, echogenic structure in the endometrial cavity up to the cervix with fluid around the endometrial cavity showed evidence of pyometra
[15]	29	Abdominal pain and menometrorrhagia	No	10 mm endometrial thickness without focal abnormalities
[16]	65	Postmenopausal bleeding	Vagina	Normal
[17]	55	Postmenopausal bleeding and painful intercourse	No	Thickened endometrium measuring approximately 20 mm in diameter
[18]	67	Vaginal bleeding	Omentum	Multiloculated, solid cystic mass up to 12 cm diameter
Present case	43	Pelvic pain	No	Acute stage: Endometrial cavity and cervical canal distended by anechoic endocavitary fluid collection and irregular thickened heterogeneous endometrium. Chronic stage: Hypoechoic thickened endometrium, hyperechoic internal myometrial layer; irregular endometrium-myometrium junction.

In a case report of a 55-year-old woman examined for postmenopausal bleeding and painful intercourse, an ultrasound examination described a thickened, indistinct endometrium, measuring approximately 20 mm in diameter. Otherwise, the examination results were normal. In this case, sonohysterography confirmed the endometrial abnormality though the imaging appearance was nonspecific [17]. In a case report of a 64-year-old postmenopausal female with a history of mucoid to foul‐smelling white vaginal discharge, an ultrasound exam revealed an enlarged uterus of 10.7 cm × 7.5 cm filled with linear, echogenic structure in the endometrial cavity up to the cervix with fluid around. The endometrial cavity showed evidence of pyometra [14]. There is also a case in the literature of a 29-year-old gravida 3, para 2-1-0-2, with a history of anovulation evaluated for abdominal pain and menometrorrhagia of five months’ duration. The transvaginal ultrasound showed a 10 mm endometrial thickness without focal abnormalities [15]. In common with these cited cases, we described the presence of an irregular heterogenous thickening of the endometrium [14,15,17]. In addition, we tracked the evolution of the ultrasound features in endometrial malacoplakia, describing two distinct ultrasound findings: anechoic endocavitary fluid distending the endometrial cavity with irregular heterogeneous thickening of the endometrium during the initial acute stage, followed by hypoechoic endometrium with a hyperechoic internal myometrial layer in the following chronic stage of the disease.

To date, ultrasound is not considered a reliable diagnostic tool for malacoplakia, as the condition typically presents as a mass or lesion that can mimic other benign or malignant growths and as imaging findings of malacoplakia can vary depending on the location and extent of the disease. However, ultrasound findings could represent an initial screening tool to identify any abnormal findings, such as thickening of the endometrium, abnormal intracavitary fluid collections, and the presence of masses or nodules within the endometrium that may require further investigation. Malacoplakia should be considered in the differential diagnosis of menstrual abnormalities and in the future, knowing the ultrasound characteristics of malakoplakia could help to better and earlier identify this rare disease.

The presence of fluid in the uterus, also known as endometrial fluid, is a non-specific finding on ultrasound and can be caused by various factors such as hormonal changes, infection, inflammation, or tumors, so the presence of fluid in the uterus alone is not a definitive sign of this condition.

Endometrial malacoplakia is a rare condition with only 15 cases reported in the medical literature to our knowledge. The exact cause of the disease is not fully understood, but recent research suggests that there is a problem with the way macrophages digest bacteria [2,20]. Immunodeficiency conditions, such as steroid use, diabetes, primary immunodeficiency, and AIDS, have been linked to various complications after cesarean section such as malacoplakia and many more [21-23]. When the phagolysosomal activity of macrophages is compromised due to immunodeficiency, bacteria are not fully digested and Michaelis-Gutmann (MG) bodies form. These bodies are a characteristic feature of malacoplakia, but they may not be present in the early stages of the disease. We describe the first case of a patient developing endometrial malacoplakia following a cesarean section. Although bacterial contamination may have contributed to the development of the disease, the patient did not have a known immunodeficiency condition. It is possible that cytokines released by the placental cells during pregnancy, which play a role in regulating the immune system and promoting maternal-fetal tolerance, may have been involved in the development of the disease [24,25]. Diagnosis is challenging and based on a combination of clinical presentation, imaging studies, and histological examination of affected tissues. A careful differential diagnosis is necessary in order to rule out other inflammatory processes and uterine neoplasms, as the condition typically presents as a mass or lesion that can mimic other benign or malignant growths. If malacoplakia is suspected, a biopsy of the affected tissue is typically performed to confirm the diagnosis and rule out other possible causes of the observed changes [26]. Our case report is the first that describes the evolution of 2D and 3D ultrasound and histological features, which can help clinicians suspect this rare disease. In general ultrasound findings that suggest the possible presence of endometrial malacoplakia are irregular thickening of the endometrial lining; hyperechoic inhomogeneous internal layer of the myometrium; and the presence of masses, nodules, cystic areas, or anechoic fluid within the endometrium. In our report, we found that these features vary depending on the acute or chronic stage of inflammation of malacoplakia. During the acute stage, we found an endometrial cavity and cervical canal distended by an anechoic endocavitary fluid collection and inhomogeneous hyperechoic thickening of the endometrium (possible hyperemia, tissue edema or increased blood flow of the inflamed areas using color doppler can also be reported). We did not report mass-like lesions, but these can also be found given the nodular nature of malacoplakia and can lead to misdiagnosis as tumors when present. Later in the chronic stage of the disease, we reported an increased myometrial echogenicity due to the presence of collagen deposition, since long-standing inflammation processes lead to fibrotic changes, and loss in the normal architecture of the endometrium-myometrium junction, resulting in inhomogeneous hypoechoic endometrium, inhomogeneous hyperechoic internal myometrium, and irregular endometrium-myometrium junction on ultrasound. In our case report the main ultrasound finding that persisted during all stages of the endometrial inflammation was the presence of a fluid collection in the uterine cavity, with echogenicity varying depending on the stage of evolution of the inflammatory process. It is important to note that these findings are not specific to endometrial malacoplakia and can also be seen in other conditions such as endometrial hyperplasia or endometrial cancer. Therefore, a definitive diagnosis of endometrial malacoplakia requires histological examination of the affected tissues. If malacoplakia is suspected, additional imaging studies, such as a CT scan or MRI, may be performed to confirm the diagnosis and determine the extent of the disease. These techniques may be used in conjunction with ultrasound or other imaging modalities to improve the specificity and sensitivity of diagnosis [24,27]. In conclusion, the diagnosis of malacoplakia is based on a combination of clinical presentation; imaging studies, such as ultrasound; and histological examination of affected tissues. Endometrial malacoplakia typically presents with postmenopausal bleeding, abnormal uterine bleeding in menstruating women, or a suspicious uterine mass [10-27]. Our case is the first of malacoplakia presenting with abundant vaginal sero-hematic secretions months after surgery for a cesarean section. Treatment for malacoplakia involves both surgical and medical options, but there are no established guidelines. The type of surgical intervention varies depending on the site and extent of the disease. Antibiotic therapy is mainly focused on drugs that can reach high concentrations within macrophages, such as quinolones or trimethoprim-sulfamethoxazole. Currently, a combination of antibiotics and surgery is the most effective treatment protocol. A new protocol involving antibiotics, bethanechol, and ascorbic acid has been used in the treatment of cerebral malacoplakia [20,26,27]. In the case we report, the patient was treated with uterine curettage and quinolone antibiotics, resulting in a rapid clinical improvement with complete resolution of symptoms and a normal gynecological ultrasound report on all follow-up exams performed up to date. During the last follow-up in 2023, the only symptom reported was secondary amenorrhea. Although the management of this rare condition is challenging, the prognosis is generally good. However, recurrence and complications may occur over time. Ultrasound can also be a useful and affordable clinical tool for follow-up in patients with malacoplakia. As research in the field continues to evolve, new ultrasound techniques and protocols may be developed that can aid in the diagnosis of malacoplakia.

## Conclusions

Overall, while ultrasound findings alone are not currently sufficient to diagnose malacoplakia, they may become part of a more comprehensive diagnostic approach in the future. Further research is needed to evaluate the potential of these techniques and to develop more specific and sensitive diagnostic tools for malacoplakia.
